# Assessing the impact of ETS trading profit on emission abatements based on firm-level transactions

**DOI:** 10.1038/s41467-020-15996-1

**Published:** 2020-04-29

**Authors:** Jianfeng Guo, Fu Gu, Yinpeng Liu, Xi Liang, Jianlei Mo, Ying Fan

**Affiliations:** 1 Institutes of Science and Development, Chinese Academy of Sciences, 100190 Beijing, China; 2 School of Public Policy and Management, University of Chinese Academy of Sciences, 100049 Beijing, China; 3 Department of Industrial and System Engineering, Zhejiang University, Hangzhou, 310027 China; 4 National Institute of Innovation Management, Zhejiang University, Hangzhou, 310027 China; 5 Centre for Business and Climate Change, The University of Edinburgh, Edinburgh, EH8 9JS UK; 6 School of Economics and Management, Beihang University, 100191 Beijing, China

**Keywords:** Climate sciences, Environmental social sciences, Social sciences

## Abstract

The EU Emission Trading System (ETS) is the oldest and currently the largest carbon market in the world, but its purpose of stimulating carbon emissions via trading profits remains unexamined. Based on the complete firm-level transaction records of the EU ETS Phases I and II, here we show that the participating firms’ trading profits and their emission abatements are positively correlated, and the correlation becomes stronger in Phase II than Phase I. Specifically, we observe that non-linearity exists in the correlation; higher firm-level emission abatements can realize larger trading profits. This pattern affects the market fairness, though it may be helpful to incentivise emission abatements. The correlation is more regulated in Phase II than it is in Phase I, thereby indicating that the Phase II is more mature. We also observe that the state-level abatements are largely driven by industrial giants.

## Abstract (web summary)

Carbon emission trading is an important market-based policy instrument to reduce GHG emission using reward-punishment mechanism. Here the authors show that the EU emission trading schemes operate at its designed purpose and there is a positive and linear relationship between firm profits and the firms’ efforts in abatement.

## Introduction

Carbon emission trading has been proposed as a market-based policy instrument to reduce greenhouse gas (GHG) emissions (mainly CO_2_) at the least possible cost. The European Union (EU) launched the European Union Emission Trading Scheme (EU ETS) on January 1, 2005 to fulfill the commitment of 8% reduction in GHG emissions by 2012 compared with 1990 proposed in the Kyoto Protocol^1^. Following the EU ETS, a series of carbon markets have been established successively, including the Regional Greenhouse Gas Initiative in the United States and the Pilot Carbon Trading Markets in China^2,3^. However, whether the incentives offered by such markets can be realized in emission abatements needs to be examined, because similar markets are continuously implemented worldwide^2–5^.

The EU ETS is a popular topic in the carbon market literature^6–26^ because it is the oldest and currently the largest carbon market in the world. The EU ETS covers over 11,000 installations from various industrial sectors across all EU member states and even non-EU countries such as Norway, Iceland, and Liechtenstein^2,3^. It is a cap-and-trade system, where a finite quantity of emission permits, namely the Eurpean Union Allowances (EUAs), is granted to the participating firms. There also exist two other tradable permits, i.e., the Emission Reduction Units (ERUs) and the Certified Emission Reductions (CERs). Firms report their annual carbon emissions to the authorities and surrender equivalent permits, each of which entitles its holder to emit 1 tonne of CO_2_. The surpluses or shortages of the permits are related to the quantity of one’s abatements for trading, thereby influencing the firms’ tradings.

Previous studies investigated various aspects related to the operation of the EU ETS, including market efficiency^7–9^, allowance allocation^9–11^, pricing mechanism^12–20^, trade frictions^21^, and market stability reserve^22^. Moreover, the EU ETS has an influence on stock markets^23–26^, energy markets^27–31^, and energy-intensive industries such as power^32^, metallurgical^33^, and transport industries^34,35^. However, little effort has been exerted on the incentiving effect of allowance trading on abatements; the market incentives are supposed to motivate the emitters to spontaneously and continuously reduce their GHG emissions. To date, this issue has never been quantitatively investigated, even though the EU ETS has been operating for over a decade.

The realization of the market incentives of the EU ETS is supposed to be guaranteed by two major safeguards. First, the abatements for trading are positively correlated to trading profits; higher profits ought to be acquired from more carbon abatements, while higher expenses shall be paid due to insufficient abatements. Since emission reduction is a long-term compaign^3,36,37^, firms should achieve abatements continuously, and the cap-and-trade market would provide incentives to support such efforts. Second, market regulation aims at constraining potential manipulation of dominant participants, thereby ensuring market fairness. Although cumulative market incentives can stimulate firms to abate their emissions continuously, it has potentials to endow big players with the market power, which could jeopardize the market fairness. However, to the best of our knowledge, the effectiveness of these safeguards remains unexamined.

Focusing on the subjects of the Phase I and II, this study examines the effectiveness of the market incentives that aims at stimulating abatements for trading. In detail, we reviewed the correlation between first the profits of firms (i.e., emitters) acquired from trading physical carbon allowances (defined as trading profits and denoted as profits *r*), and second the emission abatements for trading (the gaps between the allocated (and obtained from auctions, i.e., baseline emissions) and the surrendered allowances (i.e., actual emission), denoted as gap *g*), in a complete phase. Different types of abatements are illustrated in the Supplementary Discussion: Decomposition of emission abatements.

We aggregate the cash flows of all physical transactions of EUAs, CERs, and ERUs at their corresponding daily prices. The resulting values of the cash flows are considered as the trading profits (i.e., profits from allowance trading). Although the use of the daily values may cause deviations in the daily cash flows of individual firms, such deviations would not affect the cash flow distribution of all firms in a complete phase. The same principle is applicable for the daily-based stock market analysis. Therefore, the use of daily carbon prices does not affect the outcomes of this research, though using high-frequency prices could improve the accuracy of cash flow calculation. The data of the allocated and surrendered allowances are obtained from the compliance records. The impact of the firms’ characteristics and other external factors, such as macroeconomics, are excluded, since such factors are irrelevant to our objective of examining the impact of incentives on abatements.

The contribution of this study is fourfold. First, based on the complete firm-level trading records of Phase I and II in the EU ETS, we observe that emission abatements and trading profits are generally positively correlated. The effectiveness of the market incentives has been empirically verified, as abatements can be realized into profits. Second, using a quantile regression, we find the presence of non-linearity in the positive correlation between trading profits and emission abatements. Higher abatements have been realized greater profits; the Matthew effect (i.e., the rates of return of participants are positively correlated to their profit levels) exists in the EU ETS. Despite the pattern being helpful in stimulating carbon abatements, it grants big players with manipulative market power, thereby damaging the market fairness. Third, our findings suggest that the EU ETS is more mature in Phase II than in Phase I, as the incentives were given to participants in a fairer manner; the positive coefficient between *r* and *g* becomes smaller in Phase II than that in Phase I. Moreover, in Phase II, the extreme trading patterns are controlled and the Matthew effect is weakened. Lastly, we find that industrial giants play a decisive role in determining the state-level correlation between trading profits and emission abatements. Since big firms have higher rates of return in the allowance trading, their abatements and tradings require specific attention.

## Results

### Correlating profits and abatements

**Fig. 1:**
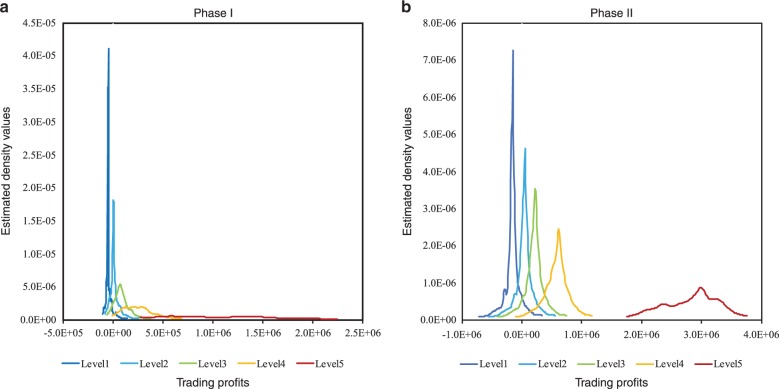
Comparison of the profit distributions in different quantiles based on the firm-level trading data. Five distributions (line Level 1 to Level 5) are presented with trading profits of the 0.10, 0.30, 0.50, 0.70, and 0.90 quantiles. The densities of the distributions are calculated by the estimated conditional quantile. **a**, **b** The profit distributions of Phase I and II of the EU ETS, respectively.

Figure 1 presents the complete quantile distribution. It shows that the density function shifts as the conditional quantile increases, which is evidence that the relation between *r* and *g* is non-linear. The presence of location shift proves that the relationship can be modeled by the quantile regression.

Table 1 summarizes the descriptive statistics of the estimated *r* of firms with different *g* quantiles. There are four observations on firm-quantiles relationship. First, the mean values of *r* increases as the values of *g* increases, thereby indicating that higher gaps correspond to higher profits. In other words, GHG abatements are financially rewarded in the cap-and-trade system. Second, as *g* increases, the standard deviation increases together with a decrease of the Kurtosis value. These results can be explained by a wide positive gap, which exerts low pressure on the firms to surrender allowances, thereby providing firms with diversified options to trade their extra allowances. Consequently, the range of the trading profits of firms tend to be wider than those of firms with narrow gaps. Third, the skewness values are positive in all the quantiles, thereby indicating that only a few firms can profit more highly than their sample average. This phenomenon is considerably significant in Phase I. Fourth, the Kurtosis value decreases with increasing gap level, thereby indicating that firms with wide gaps apparently obtain their profits unevenly.

**Table 1:** Definitions and statistics of the quantiles.

Level	*τ*	Phase I					Phase II				
		*r*	*M*	*SD*	*K*	*S*	*r*	*M*	*SD*	*K*	*S*
1	0.1	−10,306.5	1.7E + 05	2.2E + 06	134.5	9.8	−12,889	7.9E + 04	4.1E + 06	89.7	1.3
2	0.3	−316.0	2.3E + 05	2.2E + 06	133.3	9.8	4,170.6	1.2E + 05	4.1E + 06	89.3	1.3
3	0.5	11,952.5	3.0E + 05	2.2E + 06	131.7	9.7	18,694.0	2.8E + 05	4.1E + 06	89.1	1.2
4	0.7	40,120.7	4.7E + 05	2.3E + 06	127.5	9.5	51,096.4	6.5E + 05	4.1E + 06	88.5	1.1
5	0.9	186,752.6	1.3E + 06	2.4E + 06	100.4	8.0	247,575.2	2.9E + 06	4.2E + 06	84.4	0.5

Level 1 firm is at the 0.1 quantile of the distribution of ***g***. Levels 2–5 are defined in a similar manner. The *M*, *SD*, *K*, and *S* denote the mean, standard deviation, kurtosis, and skewness, respectively, of the estimated ***r*** of firms with different ***g*** quantiles.

**Fig. 2:**
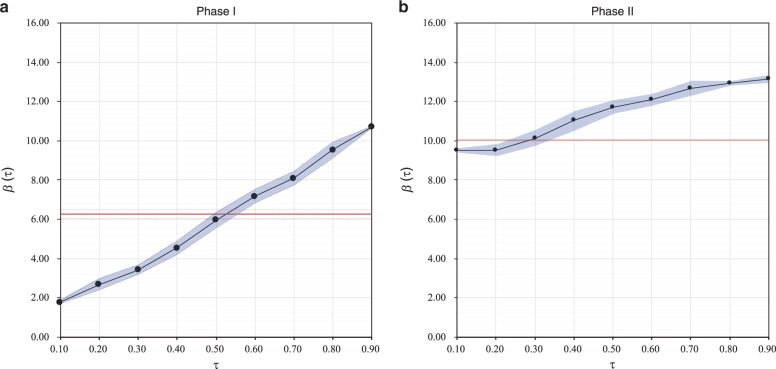
The estimated results of the linear quantile regression model based on the of the firm-level trading data. In the plots of Phases I (**a**) and II (**b**), the estimated slopes of quantile 0.1 to 0.9 are plotted as a function of ***β***(***τ***), the estimated intercepts of the results are not reported for simplicity and specificity reasons. The area enclosed by gray dotted lines represents a 95% confidence interval of estimated ***β***(***τ***). The red line indicates the overall linear coefficient and its 95% confidence interval is enclosed by red dotted lines.

This quantile regression results (Fig. 2) unveils three major findings about the effect of market incentives on emission abatements in the EU ETS. First, the coefficients of ***β***(***τ***) are above zero in all quantiles, as well as in both phases, thereby indicating that abatements for trading can be well-realized as trading profits; it is common in other forms of market. Second, the estimated coefficients of ***β***(***τ***) are greater in the higher quantiles than those in the lower ones; the presence of the Matthew effect is observed in the EU ETS. This observation shows that higher marginal profits go hand in hand with higher abatements, the realization of trading profits for emitters of different abatement levels is non-linear. Third, the increment of ***β***(***τ***) is approximately linear in Phase I, but it becomes an S-shaped trend in Phase II. In addition, the range of ***β***(***τ***) is much wider in Phase I than in Phase II. This difference implies that the Matthew effect is stronger in Phase I, while such effect is weakened in Phase II. Although promoting more abatements tends to be generally preferable, such unlimited markets incentives are not fair; advantageous emitters tend to gain the manipulative market power, thereby heavily influencing the allowance transactions. Under this consideration, the abatement motivations of the other emitters could be weakened, as they cannot acquire sufficient mariginal profits from allowance trading. On the contrary, the market becomes more mature and fair, provided that the effect of market incentives is moderate.

**Fig. 3:**
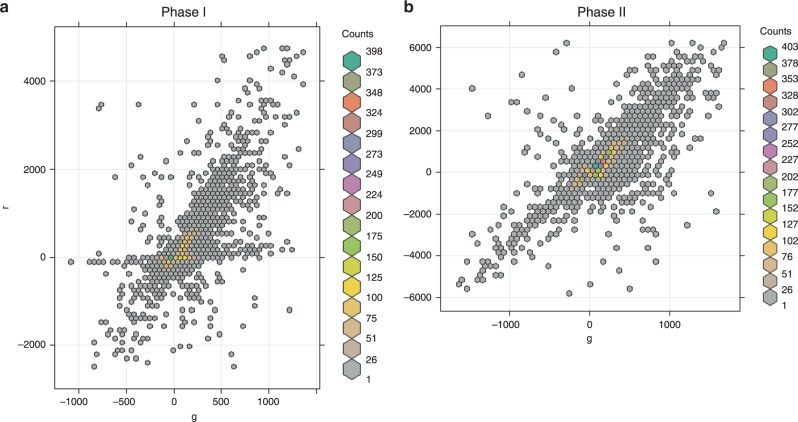
Heat maps of firms’ trading performance based on the firm-level trading data. **a**, **b** The firms’ trading performance in Phase I and Phase II, respectively. The firms are plotted on the *g*–*r* coordinate system using their processed abatements for trading *g****’*** and trading profits *r****’*** as coordinates. The different colors of the grids denote different volumes of the participating firms within the corresponding grids.

In the processed *g*–*r* coordinate system, the distribution of the firms’ coordiantes in Phase I is more dispersive, whereas firms’ coordinates in Phase II are clustered. The patterns of firms’ trading performance in the heat maps are generally compatible with the quantile regression results because the majority of the participating firms are concentrated in small linear zones around the origin of the *g*–*r* coordinate system. For Phases I and II, the distributions of the grids with more firms are similar to the overall distributions of all the participating firms. This finding indicates that the distributions of firms’ trading performance in the *g*–*r* coordinate system have multiscale fractal characteristics, thereby proving that the *g*–*r* coordinate system is robust. The linearity of Phase II is more evident than that of Phase I because the distribution of firms in the heat map of Phase II (Fig. 3b) is considerably linear and symmetric. Figure 3 provides further evidence to support the claim that EU ETS gradually reaches maturity in Phase II, as non-linearity in the realization of emission abatements is reduced. It implies that the extreme *r*–*g* patterns are more regulated in Phase II. Based on the firm-level empirical analysis, we confirm that the incentiving effect is valid and the market has matured.

### Trading profits at state-level

**Fig. 4:**
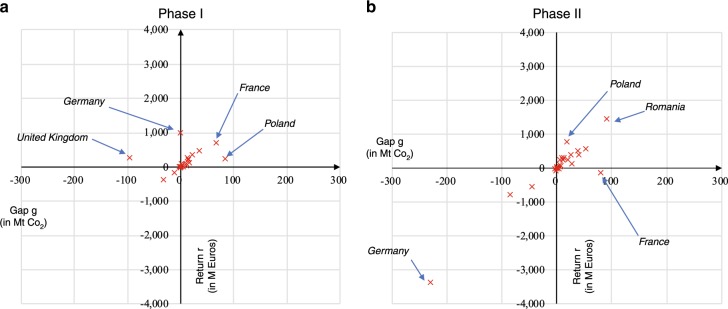
State-level trading performance based on the aggregation of the firm-level trading data. The participating states are plotted on the *g*–*r* coordinate system, where the combined *g* and *r* of all firms that belong to one state are employed as the coordinates of the state. States with abnormal trading pattern (i.e., states with *g–r* coordinates that are not in the generic cluster) include the UK, Germany, France, Poland, and Spain in Phase I (**a**); and Germany, France, Poland, and Romania in Phase II (**b**). Generic cluster refers to the linear sector where the coordinates of the majority of the states concentrate.

To further examine the incentivizing effect of the cap-and-trade system, we investigate the effect of market incentives on carbon abatements in the allowance trading of each EU ETS member state. Figure 4 summarizes this correlation of all participating states.

**Fig. 5:**
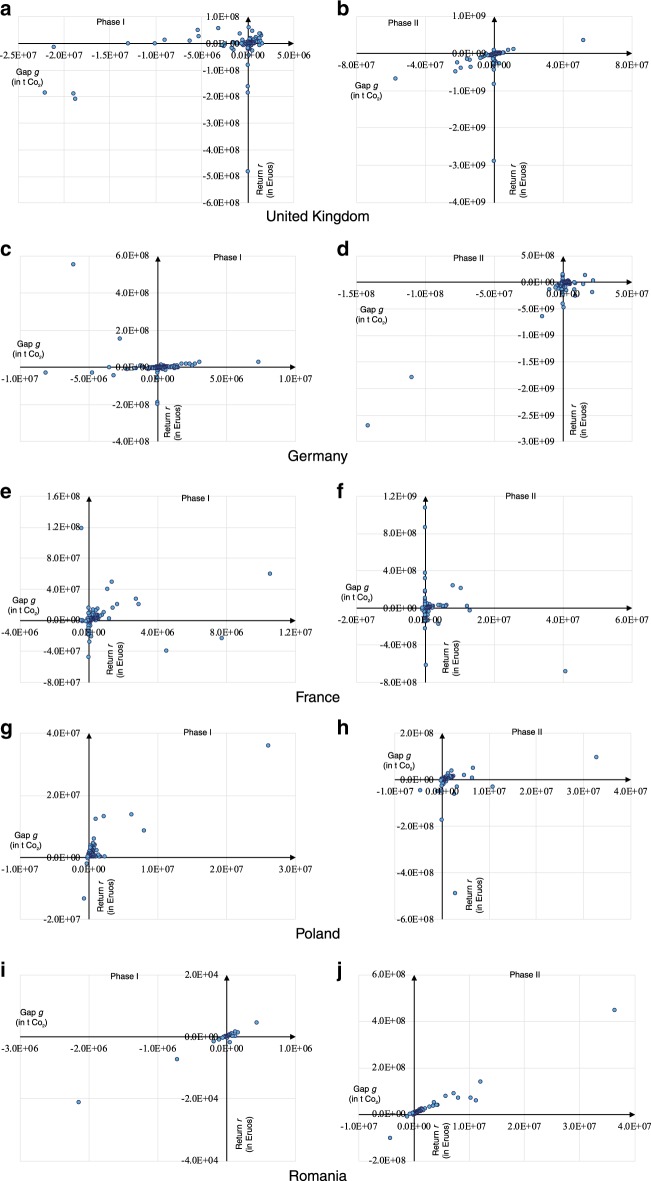
State-level trading performance of the abnormal states. By grouping the firm-level trading data into states, there are five states, namely United Kingdom, Germany, France, Poland, and Romania, abnormally performed in the EU ETS. The Phase I performance of these states is plotted on the left side of the figure (**a**, **c**, **e**, **g**, and **I**, respectively). The Phase II performance is plotted on the right side (**b**, **d**, **f**, **h**, and **j**, respectively).

Figure 4 shows that the majority of the participating states are well-ordered, whereas several abnormal states (i.e., states that are not in the clustered part of the linear sector) are highlighted. In Phase I, the UK had positive trading profit with a negative allowance gap. Germany made the highest profit, but its abatement was nearly zero. France gained an increased positive trading profit from its positive abatement. Poland profited slightly from the market, although it had the highest abatement. Spain had the lowest trading profit and allowance gap. In Phase II, Germany held both the lowest trading profit and emission abatement. France had the second-highest abatement but recorded a trading loss from the EU ETS. Poland obtained high trading profit at low emission abatement. Romania gained the highest trading profit and the largest emission abatement. However, the state-level trading profits and emission abatements of the majority of the states have positive linear correlation. Therefore, we can classify these abnormal states into two categories: first states with deviated performance (i.e., the UK, Germany, and Poland in Phase I; and Germany, France, and Poland in Phase II); and second states with extreme trading patterns (i.e., France and Spain in Phase I, and Romania in Phase II). Figure 5 depicts the firm-level *g–r* correlations of these abnormal states.

Figure 5 reveals that the *g–r* correlations of the majority of the firms in these states in both phases are linear and positive. On the contrary, the state-level *g–r* correlation is disturbed by some firms with increased trading profits or wide gaps. In other words, the national trading patterns of the participating states in EU ETS are mainly determined by several domestic industrial giants because their *r* and *g* values are much higher than those of others. The quantile regression results (Fig. 2) can be attributed to the trading patterns of the participating firms because the majority of these firms behave in a similar manner. By contrast, only a few big firms contribute to most of the observed deviations.

These abnormal giants have three distinctive patterns. First, the distribution of the *g–r* correlations of these firms is relatively dispersive, thereby disturbing the state-level trading patterns. Typical examples include the UK in Phase I, Germany in Phase II, and France and Poland in both phases. This observation implies that firms did not accurately predict their emission levels or allowance values. Second, firms with high trading volumes amended the trading pattern of the state, even if the *g–r* correlations of most firms in the same state are abnormal. Germany in Phase I fits this scenario. We posit that firms with advanced low-carbon technologies have low expectations of allocated allowances. Moreover, only a few distinguishable firms have the chance of profiting from carbon allowance trading. Third, although the *g–r* coordinate of firms within the same state are generally linear, some participating firms evidently deviate from the general cluster. One typical example is Romania in Phase II.

In summary, the number of abnormal firms is limited and their distribution appears to be random and symmetric in EU ETS. This result implies that the cap-and-trade system is generally effective in reducing GHG emissions. The *g–r* cooridantes of the majority of the participating firms concentrate in the linear sector, thereby proving the high effectiveness of the incentivizing effect of the cap-and-trade carbon market. The trading profits of a single firm are determined by the gap of carbon allowances but are also under the influences of the decisions or characteristics of firms, such as market expectations and trading strategies. Our analysis suggests that firms’ trading profits are positively correlated with emission abatements for trading but will deviate owing to the presence of other influencing factors.

## Discussion

Our analysis provides three important findings based on the unique complete firm-level transaction data set. First, the incentivizing effect of carbon allowance trading of the EU ETS is realized; we observe that the total trading profits of firms are positively correlated with their carbon abatements for trading. This finding reveals that as a market-based instrument to reduce carbon emissions, the cap-and-trade system is generally effective, even though large allowances were simply traded for compliance obligation^3,12^.

Second, we find that non-linearity exists in the correlations between trading profits ***r*** and emission abatements for trading ***g***. This observation indicates that the firms of higher emission abatements can acquire greater profitability for carbon allowance trading; the presence of the Matthew effect in the allowance trading in the EU ETS is detected. Some participants have higher rates of return in both directions, thereby affecting the fairness of realizing the financial awards of emission abatements in the cap-and-trade market. This can be attributed to the inactive trading environment where the participants with bigger trading scale intentionally or unintentionally manipulate the market to some extent^38^. Yet, this pattern seems to be weakened in Phase II over Phase I, confirming that the EU ETS becomes more mature in the latter phase. This finding agrees with the extant macroeconomic literature that suggests the increasing maturity of EU ETS^7,8^. Stable value realization of emission abatements facilitates the financial management of carbon allowances, which are widely recognized as financial assets^39–44^ that influence a series of energy commodities such as crude oil and natural gas^29^.

Our final observation was obtained in the comparison of the state-level *r–g* correlations of the participating states in the carbon market. The majority of the states and firms have similar trading patterns because they profited proportionally to their abatements for trading. Meanwhile, certain companies with significant allowance gaps affected their countries significantly. For example, in Phase I, the UK had positive trading profit with a negative *g*, while Germany made the highest profit with a zero abatement for trading. The extant literature has reviewed the impact of EU ETS on the industrial sectors^32–35^. To the best of our knowledge, insufficient attention has been directed at the realization of national carbon abatements through firm-level trading in the scheme possibly owing to limited data availability. Accordingly, our unique firm-level trading data set fills in this knowledge gap. A direct implication of this finding for policy makers and administrators is that increased attention should be provided to monitor the transactions of these industrial giants. But this finding may be inappropriate to regulate firms’ trading behaviors in such a market. The abnormal firms may have at least one of the following features: conducting a few transactions and odd trading behaviors, e.g., conducting transactions only in certain periods. These transactions cause these firms to be overrewarded or overpunished. Hence, active and balanced transactions with directions (i.e., buy or sell) and timepoints selected based on market expectationn’ s can guarantee that firms are properly rewarded according to their gaps, thereby possibly improving the carbon abatement mechanism of the EU ETS. Among all the potential national differences that affect trading, the development of low-carbon technologies varies from one member state to another is worthy of in-depth investigation because of its influence on the majority of the participating firms at the state level.

## Methods

### Data aggregation

Our analysis is based on a unique firm-level data set that combines all transaction records in the Community Independent Transaction Log (CITL) of Phase I and the European Union Transaction Log (EUTL) of Phase II or the Kyoto Phase. The detailed aggregation procedure of this unique data set is presented in the Supplementary Method: Construction of firm-level trading data set. This unique data set can be regarded as the complete transaction log of Phases I and II allowances (. Allowance transaction data have been maximized in the microeconomic literature to identify the determinants of an emitter’s trading volume^45^, trading threshold^46^, trading activities^47^, effects of transaction costs^48^, and performance^49^. To the best of our knowledge, no research has constructed firm-level trading data in such comparable detail and so completely. Moreover, the data employed in the extant literature either only cover Phase I^45,47,48^ or fails to link installations to firms^45,49^. Using the unique microlevel trading data set, we aim to examine the effect of the market incentives that are provided by the cap-and-trade system on carbon abatements.

### Model construction

To avoid the inequality of the data and find some potential non-linear relationships, we employ a quantile regression model to investigate the correlation between firm profits(*r*) and quantiles(*g*) in EU ETS:

1 $$Q_\tau (r|g) = \alpha (\tau ) + \beta (\tau ) \times g,$$

where *α*(*τ*) is the intercept and *ß*(*τ*) is the quantile coefficient that depends on *τ*. The model is derived by specifying the *τ*th conditional quantile as *Q*_*τ*_ (*r* | *g*) and estimating *α*(*τ*) and *ß*(*τ*) as the solutions to the following equation^50^:

2 $$\min _{\alpha (\tau ),\beta (\tau )}\mathop {\sum}\limits_{i = 1}^n {\rho _\tau } \times \left[ {r_i - \alpha (\tau ) - \beta (\tau ) \times g_i} \right],$$

where *ρ*_*τ*_(*z*) is a linear loss function defined as *z*(*τ*−1) if *z* < 0; otherwise, *z*_*τ*_.

We use 20 quantiles (i.e., 0.05, 0.10, 0.15,…, 0.95) in the analysis. All regression results for these quantiles are significant for the carbon trading data of Phases I and II can be found in Fig. 2a, 2b. The quantile regression results are based on the firm-level dataset. In addition, we employ heat maps to visualize the distributions of *r* and *g* in detail. The differences in the goodness-of-fit values of the different phases can be effectively explained visually via the heat maps (Fig. 3), in which *r* and *g* are processed as follows:

3 $$g\prime = sig(g)\sqrt g ,$$

4 $$r\prime = sig(r)\sqrt r ,$$

5 $$sig(x) = \left\{ \begin{array}{l}1,x > 0\\ 0,x = 0\\ - 1,x < 0\end{array} \right.,$$

where *sig*(*x*) is a signal function.

The process and outcomes of robustness check is given in the Supplementary Method: Robustness check.

### Reporting summary

Further information on research design is available in the Nature Research Reporting Summary linked to this article.

## Supplementary information

Supplementary Information

Reporting Summary

## Data Availability

The data sets generated during or analyzed in this study are available from the corresponding author upon any reasonable requests. The raw data that our firm-level trading data were derived from are available in the public domain: EUTL dataset (https://ec.europa.eu/clima/ets/). The firm-level data and the sub data in major countries are available on Figshare (10.6084/m9.figshare.12034482.v1; 10.6084/m9.figshare.12034503.v1; 10.6084/m9.figshare.12034479.v2).

## Code availability

The data, R codes of the statistical analysis, and the results are available from the corresponding author upon any reasonable requests.
