# Supplementary material for: Assessing the impact of ETS trading profit on emission abatements based on firm-level transactions
Source: Nat Commun. 2020 Apr 29;11:2078. doi: 10.1038/s41467-020-15996-1 (PMC7190651; doi:10.1038/s41467-020-15996-1)
Supplement: Supplementary file 1 — Supplementary Information [file 41467_2020_15996_MOESM1_ESM.pdf]

Supplementary: Assessing the Impact of ETS Trading Profit on Emission Abatements based on Firm-level Transactions

Jianfeng GUO[1,2], Fu GU[3,4*], Yinpeng LIU[1,2*], Xi LIANG[5*], Jianlei MO[1,2], Ying FAN[6]

Supplementary Discussion: Decomposition of emission abatements

As indicated by the term of cap-and-trade, the promotion of GHG emission abatements via the EU ETS consists of two segments: (1) The cap is the compulsory regulative constraint that requires all participants to commit a below-baseline value of carbon emissions. (2) The trade denotes a means of exchange through which the emitters can get profits or allowances. Therefore, the total abatements can be divided into two parts: the required abatements and the abatements for trading. Supplementary Fig. 1 illustrates the decomposition of the total abatements. As previously stated, the abatements for trading can be calculated as the differences between the allocated and surrendered allowances, being defined as the allowances gap g. The gap is supposed to be positively correlated to the market incentives that awarded to the emitters for carbon emission abatements. Following the same principle, the unrealized abatements, i.e. the volume of emissions that exceeds the committed level (the allocated allowances), equals a negative gap g. Usually, one emitter with a negative g cannot get profits from trading in the EU ETS, because it has to purchase adequate carbon allowances for compliance purposes.

[1] Institutes of Science and Development, Chinese Academy of Sciences, Beijing 100190, China.
[2] School of Public Policy and Management, University of Chinese Academy of Sciences, Beijing 100049, China.
[3] Department of Industrial and System Engineering, Zhejiang University, Hangzhou 310027, China.
[4] National Institute of Innovation Management, Zhejiang University, Hangzhou 310027, China.
[5] Centre for Business and Climate Change, The University of Edinburgh, Edinburgh EH8 9JS, United Kingdom.
[6] School of Economics and Management, Beihang University, Beijing 100191, China.
[*] **Email**: Fu GU (gufu@zju.edu.cn), Yinpeng LIU (liuyinpeng@casisd.cn), Xi LIANG (xi.liang@ed.ac.uk)

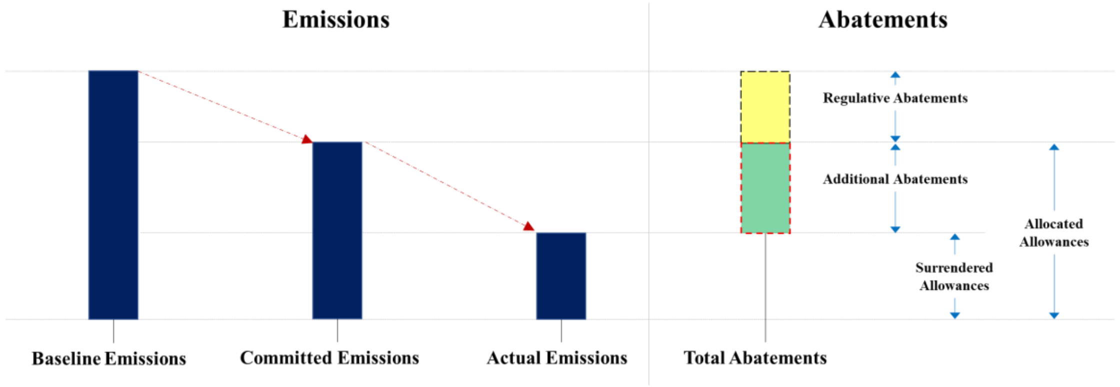

Supplementary Fig. 1 Decomposition of the total abatements. For every emitter in the EU ETS, the committed emissions are equal to the allocated allowances, and the actual emissions are equivalent to the surrendered allowances. The gap between the baseline emissions and the committed emissions are the regulated carbon abatements, which correspond to the regulated abatements. The gap between the committed emissions and actual emissions are the abatements for trading.

The incentiving effect of carbon allowance trading in the EU ETS can be intrepreted as the correlations between the emission abatements g and the trading profits r, because such correlation could explain how the emitters are rewarded based on their abatements. Notably, such correlation is not equalivent to causality. In addition, the parameter g and r can be quantified by our unique firm-level dataset of physical allowance trading.

Supplementary Method: Construction of firm-level trading data set

The CITL and EUTL data were downloaded from the European Commission's website (ec.europa.eu/environment/ets/) with lags of five and three years, respectively. Our data cover all the transaction records that occurred from January 11, 2005 (starting with the allocation of Phase I EUAs) to April 30, 2013, including the trading of the CERs issued by the Clean Development Mechanism (CDM) projects, the ERUs issued by the joint implementation projects, and general allowances in Phases I and II. EUAs were trail allowances used only in the EU ETS registries and cannot be carried through to the following phase because the Kyoto Protocol had not come into effect during Phase I. In Phase II, EUAs are converted from the assigned amount units, which are a type of allowances corresponding to the Kyoto commitment of CO_2 reduction. EUAs can only be used to cover the CO_2 emissions in the EU ETS registries.

The two types of allowance transactions are [1]: first administrative transactions, such as allocation or surrender of allowances, and second transactions between firms. Every transaction under these two categories is included with the exact price and volume, whereas the transactions between installations that belong to the same firm are merged. Therefore, the unique data set can be regarded as the complete firm-level trading log of Phases I and II of the EU ETS. The completeness of the data cannot be matched by any previous paper that only seeks to combine EUAs and CERs [2-8].

In summary, five types of carbon emission allowances have been traded during the study period: EUAs of Phase I, EUAs of Phase II, general allowances, CERs, and ERUs.

Supplementary Fig. 2 shows the coverage of the downloaded data, and the starting and end points of the five allowances within January 11, 2005 to April 30, 2013 are marked. Supplementary Fig. 2 also shows that the trading durations of the different allowances often overlap. EUAs of Phases I and II were traded near the end of Phase I. EUAs of Phase II, general allowances, CERs, and ERUs were traded simultaneously near the end of Phase II. The sum of gains or losses of trading different allowances held by one firm is thereby maximized to estimate firms' transaction performances in EU ETS.

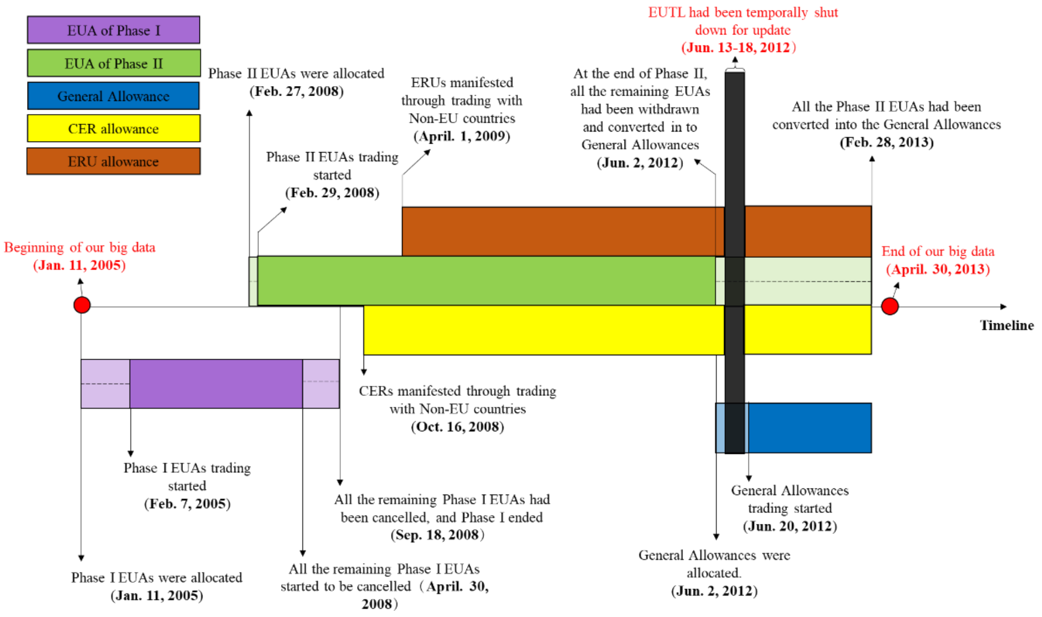

Supplementary Fig. 2 Timeline of the downloaded data. The starting and end points of the five allowances from January 11, 2005 to April 30, 2013 are marked in the vertical direction, whereas the timeline is embedded on the horizontal axis. The trading durations of the five allowances are presented in different colors. The trading duration start and end points are marked in the vertical direction, whereas the horizontal axis shows the timeline. The start and end of our data set and the shutdown week for system update are highlighted in red.

The original structure of the downloaded data is presented in Supplementary Fig. 3. Similar to the previous study [1], five groups of accounts are included in this data set: first, operator holding accounts (OHAs), which correspond to the emitting installations (emitters) in a one-to-one relationship; second, person holding accounts (PHAs), which can only hold and trade allowances; third, party accounts (PAs), which belong to the EU member states; fourth, clean development mechanism accounts (CDMAs), which trade allowances on behalf of the CDM projects; fifth, non-EU accounts (NEAs) of firms, which are unregistered in EU ETS. CDMAs and NEAs can only be found in Phase II [49]. Evidently, linking the accounts to their owners is crucial because one firm can hold several different accounts and firm's information can be modified over time. Currently, the only existing data set that links accounts to their holders is the Ownership Links and Enhanced EUTL Dataset (OLEED), which was established by the European University Institute [9,10]. However, the OLEED assigns the ownership of these accounts

by tracking the company's primary shareholder, whereas the owner and the trading decision maker are not always the same. Therefore, any analysis based on the ownership links from OLEED provides insufficient explanation on firm-level transaction data. In addition, ownerships linked in OLEED are only valid for Phase I.

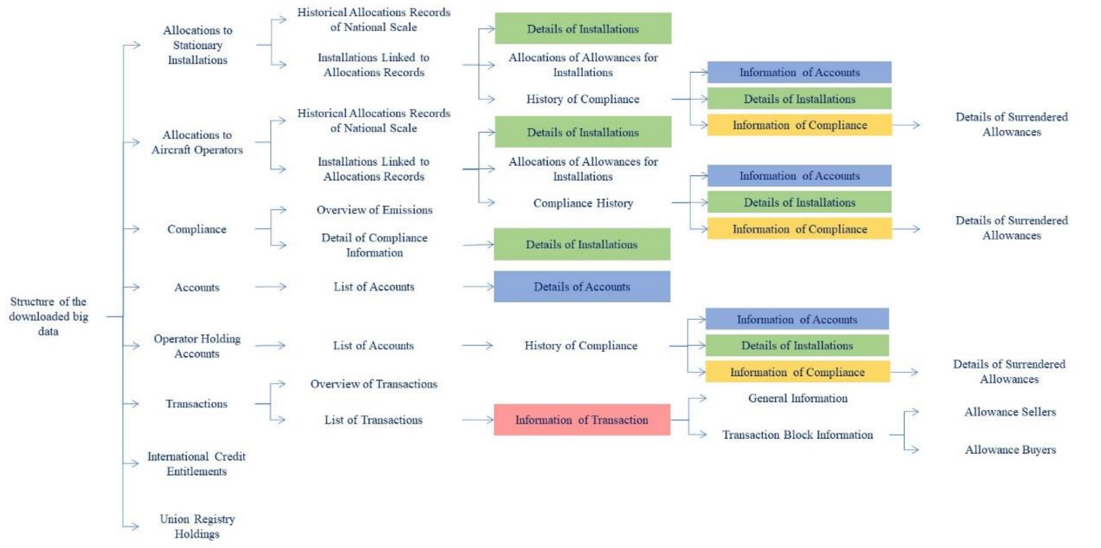

Supplementary Fig. 3 Original structure of our downloaded data. Allowance transaction-related information is stored in different categories, which are marked in distinguishable colors. The list of the pre-processing research is required before any statistical analysis.

The downloaded data are pre-processed through the following four steps to trace all the trading activities that occurred during the study period at the transaction level.

Step I: We cleaned the downloaded data and extracted four core data sets from the CITL and EUTL logs, including accounts, installations, transactions, and compliance (Supplementary Fig. 3).

Step II: We constructed a data set of companies to record the information and the accounts held by certain companies. The information of the account owners is recorded in the extracted data set of accounts. In the majority of the cases, the company's name is the ownership information. The judgment procedure is relatively simple. The firm is an emitter if it has an OHA; otherwise, the firm is a non-emitter.

Step III: We linked the transaction records to the data set of the companies according to the names of the account holders in the extracted data set of transactions. The extracted data set of transactions has 724,790 records. We validated that the transactions of EUAs of Phase I, EUAs of Phase II, CERs, and ERUs are closed within the study period. By contrast, general allowances are continuously traded at present. Therefore, surrendered allowances are equivalent to the sum of the allocated, traded, and cancelled allowances (whether compulsory or not). The accounts must exclude allowance at certain point, such as the starting and ending points of our downloaded data. Therefore, the transaction and compliance of EUAs are used in this study.

Step IV: We retrieved the carbon trading profits on the basis of the transaction records and requirement of carbon emissions based on the compliance records. Cash flow per

transaction is calculated on the basis of the transaction records of allowances and the price levels of the corresponding allowances. The price level indicators are selected on account of the market volume. The price levels of EUAs in Phase I (from January 1, 2005 to April 30, 2008) are from BlueNext, while the price levels of the EUAs, CERs and ERUs in Phase II (i.e., from December 19, 2007 to June 10, 2012) are from the Intercontinental Exchange (ICE). The arbitrage-free principle of the price spread of the different markets has limited influence on the trading profits.

The general concept of the trading profits is the summarized profits acquired from all forms of transactions, including as spots, futures, options, OTC, and swaps [49]. However, to examine the effect of the market incentives offered by the EU ETS, the profits are restricted to the cash flows aggregated from the deliverable transactions (or physical transactions) of the carbon allowances in this study. Meanwhile, undeliverable transactions, such as derivative transactions, are under the rules of financial markets and their profits should not be accounted in this research.

Supplementary Method: Robustness check

To prove the validity of applying the quantile regression, the Khmaladze test [11,12] is conducted to test the goodness-of-fit of the quantile regression model. We particularly adopt the null hypothesis that a linear model is of the location–scale shift form. The test statistics are 3.35 in the Phase I model and 1.98 in the Phase II model. Both results are below the 1% critical value of 5.56 of this test. Hence, the null hypothesis cannot be rejected and the quantile regression is acceptable for our model.

We likewise propose a robustness check to test whether the results persist when the full-sample conditions are not satisfied. We randomly select 25%, 50%, and 75% of the original data set and apply the same quantile regression model to test these data sets. Supplementary Table 1 shows that the distribution of the sampling results approximates the original results, with minor differences in their mean values and limited standard deviations. Note that when the samples account for high percentages of the original data, the results of the robustness check are increasingly close to the original results.

Supplementary Table 1. Original quantile regression results and the results of the robustness check, in which 25%, 50%, and 75% of the original data are used.

**Quantiles**	**Original results of Phase I**	**Mean values (Standard deviations) of the N% sampling results of Phase I**			**Original results of Phase II**	**Mean values (Standard deviations) of the N% sampling results of Phase II**		
		25%	50%	75%		25%	50%	75%
10%	1.78	1.85(0.81)	1.84(0.49)	1.86(0.27)	9.51	9.23(1.01)	9.30(0.57)	9.38(0.34)
20%	2.68	2.67(0.62)	2.69(0.38)	2.73(0.25)	9.53	9.62(0.84)	9.62(0.43)	9.61(0.23)
30%	3.42	3.54(0.57)	3.48(0.31)	3.45(0.17)	10.14	10.26(0.70)	10.22(0.43)	10.18(0.27)
40%	4.54	4.64(0.75)	4.58(0.45)	4.58(0.27)	11.03	10.99(0.64)	11.01(0.41)	11.03(0.25)
50%	5.96	5.97(0.78)	5.98(0.50)	6.00(0.33)	11.72	11.66(0.46)	11.70(0.27)	11.72(0.17)
60%	7.18	7.13(0.66)	7.12(0.35)	7.13(0.21)	12.09	12.16(0.39)	12.15(0.22)	12.12(0.12)
70%	8.09	8.18(0.83)	8.11(0.50)	8.07(0.30)	12.68	12.59(0.42)	12.60(0.27)	12.63(0.17)
80%	9.53	9.52(1.10)	9.51(0.69)	9.49(0.41)	12.93	12.94(0.45)	12.96(0.25)	12.96(0.13)
90%	10.71	10.64(1.28)	10.68(0.70)	10.67(0.35)	13.15	13.14(0.56)	13.15(0.30)	13.15(0.15)

Supplementary references

1 Liu, Y., Guo, J, & Fan, Y. A big data study on emitting companies' performance in the first two phases of the European Union Emission Trading Scheme. *J. Clean. Prod.* **142**, 1028-1043 (2017).

2 Marimoutou, V. & Soury, M. Energy markets and CO_2 emissions: analysis by stochastic copula autoregressive model. *Energ.*, **88**, 417-429 (2015)

3 Koenker, R. Quantile Regression. *Cambridge University Press*, Cambridge, UK (2005).

4 Chevallier, J. A model of carbon price interactions with macroeconomic and energy dynamics. *Energ. Econ.* **33**, 1295-1312 (2011).

5 Arouri, M. E. H., Jawadi, F., & Nguyen, D. K. Nonlinearities in carbon spot-futures price relationships during Phase II of the EU ETS. *Econ. Model.* **29**, 884-892 (2012).

6 Chevallier, J. Variance risk-premia in CO_2 markets. *Econ. Model.* **31**, 598-605 (2013).

7 Koop, G. & Tole, L. Modeling the relationship between European carbon permits and certified emission reductions. *J. Empir. Finance* **24**, 166-181 (2013).

8 Ortas, E. & Álvarez, I. The efficacy of the European Union Emissions Trading Scheme: depicting the co-movement of carbon assets and energy commodities through wavelet decomposition. *J. Clean. Prod.* **116**, 40-49 (2016).

9 Jaraite, J., Jong, T., Kazukauskas, A., Zaklan, A., & Zeitlberger, A. Ownership links and enhanced EUTL dataset. *European University Institute*, Florence (2013). Available at: http://fsr.eui.eu/CPRU/EUTLTransactionData.aspx

10 Jaraite, J., Jong, T., Kazukauskas, A., Zaklan, A., & Zeitlberger, A. Matching EU ETS accounts to historical parent companies: a technical note. *European University Institute*, Florence (2013). Available at: http://fsr.eui.eu/CPRU/EUTLTransactionData.aspx

11 Khmaladze, E. V. Martingale approach in the theory of goodness-of-fit tests. *Theory Prob. Appl.* **26**, 240-257 (1982).

12 Koenker, R. & Xiao, Z. Inference on the quantile regression process. *Econometrica* **70**, 1583-1612 (2002).
