# Supplementary material for: Assessing the impact of ETS trading profit on emission abatements based on firm-level transactions
Source: Nat Commun. 2020 Apr 29;11:2078. doi: 10.1038/s41467-020-15996-1 (PMC7190651; doi:10.1038/s41467-020-15996-1)
Supplement: Supplementary file 2 — Reporting Summary [file 41467_2020_15996_MOESM2_ESM.pdf]

nature research

Corresponding author(s): Fu GU, Yinpeng LIU, Xi LIANG

Last updated by author(s): 2020-4-4

Reporting Summary

Nature Research wishes to improve the reproducibility of the work that we publish. This form provides structure for consistency and transparency in reporting. For further information on Nature Research policies, see Authors & Referees and the Editorial Policy Checklist.

Statistics

For all statistical analyses, confirm that the following items are present in the figure legend, table legend, main text, or Methods section.

n/a	Confirmed	
☐	☒	The exact sample size (*n*) for each experimental group/condition, given as a discrete number and unit of measurement
☐	☒	A statement on whether measurements were taken from distinct samples or whether the same sample was measured repeatedly
☒	☐	The statistical test(s) used AND whether they are one- or two-sided<br>*Only common tests should be described solely by name; describe more complex techniques in the Methods section.*
☐	☒	A description of all covariates tested
☐	☒	A description of any assumptions or corrections, such as tests of normality and adjustment for multiple comparisons
☐	☒	A full description of the statistical parameters including central tendency (e.g. means) or other basic estimates (e.g. regression coefficient) AND variation (e.g. standard deviation) or associated estimates of uncertainty (e.g. confidence intervals)
☐	☒	For null hypothesis testing, the test statistic (e.g. *F*, *t*, *r*) with confidence intervals, effect sizes, degrees of freedom and *P* value noted<br>*Give P values as exact values whenever suitable.*
☒	☐	For Bayesian analysis, information on the choice of priors and Markov chain Monte Carlo settings
☒	☐	For hierarchical and complex designs, identification of the appropriate level for tests and full reporting of outcomes
☐	☒	Estimates of effect sizes (e.g. Cohen's *d*, Pearson's *r*), indicating how they were calculated

Our web collection on statistics for biologists contains articles on many of the points above.

Software and code

Policy information about availability of computer code

Data collection	The data was collect by a custom code programmed in JAVA (v1.9) and developed in MyEclipse. The data was recorded in MySql (v8.0) database.
Data analysis	The data was analyzed by a custom code programmed in R (v3.5) and developed in RStudio (v1.1) . The 'quantreg', 'hexbin', 'RColorBrewer' packages of R were used in the code.

For manuscripts utilizing custom algorithms or software that are central to the research but not yet described in published literature, software must be made available to editors/reviewers. We strongly encourage code deposition in a community repository (e.g. GitHub). See the Nature Research guidelines for submitting code & software for further information.

Data

Policy information about availability of data

All manuscripts must include a data availability statement. This statement should provide the following information, where applicable:

- Accession codes, unique identifiers, or web links for publicly available datasets
- A list of figures that have associated raw data
- A description of any restrictions on data availability

The data sets generated during or analyzed in this study are available from the corresponding author upon any reasonable requests. The raw data that our firm-level trading data were derived from are available in the public domain: EUTL dataset (https://ec.europa.eu/clima/ets/). The firm-level data and the sub data in major countries are available on Figshare (https://doi.org/10.6084/m9.figshare.12034482.v1; https://doi.org/10.6084/m9.figshare.12034503.v1; https://doi.org/10.6084/m9.figshare.12034479.v2).

Field-specific reporting

Please select the one below that is the best fit for your research. If you are not sure, read the appropriate sections before making your selection.

☐ Life sciences ☒ Behavioural & social sciences ☐ Ecological, evolutionary & environmental sciences

For a reference copy of the document with all sections, see nature.com/documents/nr-reporting-summary-flat.pdf

Behavioural & social sciences study design

All studies must disclose on these points even when the disclosure is negative.

Study description	This study focuses on the effect of market incentives on emission abatements by examining the correlation between firms' trading profits and their carbon abatements. The quantitative observational data is based on the complete firm-level transaction records.
Research sample	The research sample are the companies in the EU ETS. The demographic information includes trading details, annual emissions, and annual carbon allowances allocation. The sample covered all the participating companies.
Sampling strategy	No sample-size calculation was performed because we used a complete sample to guarantee the sample size are sufficient.
Data collection	The data is collected from the European Union Transaction Log website by a java program. The program downloads every webpage of the website, then parses sand records the data in MySql database. The data is from a real market, so the experimental condition is out of our consideration.
Timing	For the Phase Ⅰ, the data is from Jan 11, 2005 to Sep 18, 2008; for the Phase Ⅱ, the data is from Feb 27, 2008 to April 30, 2013.
Data exclusions	63 data in Phase Ⅰ data (4,505 in total) and 78 data in Phase Ⅱ data (6,237 in total) were excluded to reduce the influence of outlier. If the trading profits or carbon abatements are higher than the 99.75% quantile or lower than 0.25% quantile, then it should be excluded as an outlier. The criteria is commonly used in statistics.
Non-participation	No participants was dropped out.
Randomization	Participants were not allocated in to experimental groups.

Reporting for specific materials, systems and methods

We require information from authors about some types of materials, experimental systems and methods used in many studies. Here, indicate whether each material, system or method listed is relevant to your study. If you are not sure if a list item applies to your research, read the appropriate section before selecting a response.

Materials & experimental systems

n/a	Involved in the study	
☒	☐	Antibodies
☒	☐	Eukaryotic cell lines
☒	☐	Palaeontology
☒	☐	Animals and other organisms
☒	☐	Human research participants
☒	☐	Clinical data

Methods

n/a	Involved in the study	
☒	☐	ChIP-seq
☒	☐	Flow cytometry
☒	☐	MRI-based neuroimaging
